# The musculature and skeleton of the pelvic fin of the crocodile shark, *Pseudocarcharias kamoharai* (Matsubara, 1936) (Elasmobranchii: Lamniformes): Morphology and sexual dimorphism

**DOI:** 10.1002/ar.70201

**Published:** 2026-04-09

**Authors:** Laura F. Mianutti, Kenshu Shimada, João Paulo C. B. da Silva

**Affiliations:** ^1^ Departamento de Sistemática e Ecologia, Centro de Ciências Exatas e da Natureza Universidade Federal da Paraíba, Castelo Branco João Pessoa Brazil; ^2^ Department of Environmental Science and Studies DePaul University Chicago Illinois USA; ^3^ Department of Biological Sciences DePaul University Chicago Illinois USA; ^4^ Sternberg Museum of Natural History Fort Hays State University Hays Kansas USA; ^5^ Integrative Research Center Field Museum of Natural History Chicago Illinois USA

**Keywords:** clasper, myology, Pseudocarcharhiidae, secondary sexual characteristic, siphon sac

## Abstract

*Pseudocarcharias kamoharai* (Matsubara, 1936) (Elasmobranchii: Lamniformes) is a small, viviparous, pseudocarchariid shark distributed throughout tropical ocean waters worldwide. Although the appendicular skeleton of elasmobranchs has been explored in the literature, little is known about the clasper skeleton. Additionally, the appendicular musculature in general is not well studied in elasmobranchs, especially that of the pelvic fins and claspers. The same can be said about the siphon sac, a muscular bladder associated with the clasper and related to reproduction in male elasmobranchs. Here, we aim to expand the amount of morphological information related to the secondary sexual characteristics and to describe the pelvic musculature and skeleton, including a description of the clasper of the crocodile shark. The connected siphon sacs have a general inverted “Y” shape, with their anterior margins “M”‐shaped and their posterior portions forked. The musculature of the ventral surface of the pelvic fin in both sexes is similar. However, the musculature of the dorsal surface of the male has an extra muscle in comparison to the female, the *flexor internus*. Moreover, the clasper has a few exclusive muscles, such as the *dilatator*. In relation to the clasper skeleton, its morphology follows what is seen in other lamniforms. This study marks the first report of sexual dimorphism related exclusively to the pelvic fin musculature in Lamniformes.

## Graphical abstract

This work brings new information on the sexual characteristics of the crocodile shark (*Pseudocarcharias kamoharai*) exploring the pelvic fin musculature, as well as the siphon sac and the musculature and skeleton of the clasper. Our paper is the first to point out clearly the sexual dimorphism related exclusively to the pelvic fin musculature in males, which includes additional muscular components in relation to the females.

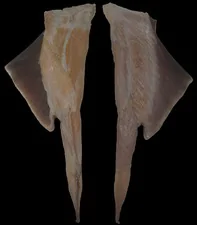

## 1 INTRODUCTION

It is well documented that male and female chondrichthyans exhibit sexual dimorphism in several morphological aspects. Some of the most common differences are the body size, in which females are frequently larger and have greater sizes at maturity than males (Colonello et al., 2020; Oliveira et al., 2010; Porcu et al., 2014; Powter & Gladstone, 2008; Semba et al., 2009), the greater thickness of the skin in females in several elasmobranch species related to their species‐specific reproductive behavior (Crooks & Waring, 2013; Hagood et al., 2023; Kajiura et al., 2000; Pratt & Carrier, 2001), or the more sharply pointed tooth cusps in males than females, which may become even more prominent during the reproductive season (Berio et al., 2020; Guzmán‐Castellanos et al., 2024; Kajiura & Tricas, 1996; Litvinov & Laptikhovsky, 2005). Another well‐known sexual dimorphism in chondrichthyans is the presence of paired siphon sacs and claspers. The siphon sac is a muscular bladder located abdominally between the epidermis and the hypaxial musculature, and it is associated with the clasper, a rod‐like sexual organ universally present in male chondrichthyans. Both structures play a key role in the reproduction of sharks; yet, they are hardly studied, especially the siphon sac.

The pelvic fins and associated structures are important for the locomotion and reproduction of elasmobranchs. Many batoids (e.g., stakes and rays) perform the punting locomotion, which helps the animal to maneuver on the substrate, find prey, and avoid predators (Macesic & Kajiura, 2010). Along with the pectoral fins, the pelvic fins also help the animal to lift its body in the water (Ebert et al., 2021). Yet, much of the anatomy of the pelvic region of elasmobranchs has focused on their skeleton (Silva, 2014; Silva et al., 2021; Silva et al., 2023; Silva & Vaz, 2023) rather than on its associated musculature (Goto, 2001; Goto et al., 1999; Macesic & Kajiura, 2010). Whereas Moreira and Carvalho (2019) examined the musculature of the clasper in some shark taxa, besides its skeleton, one notable exception is Leigh‐Sharpe's (1920) study, which explored the secondary sexual characters (e.g., siphon sac, parasiphon, and some structures of the clasper) of elasmobranch fishes and determined that the siphon sac is used to aid in flushing the spermatozoa into the oviduct of the female through the groove of the clasper by the seawater stored inside the siphon. Subsequently, Gilbert and Heath (1972) further investigated the function of the siphon sac and how a shark may fill its siphons with seawater. The observation of the use of siphon sacs by sharks in the wild or in captivity is also rare, although some examples are known (e.g., Tricas & Le Feuvre, 1985; Whitney et al., 2004).

Lamniformes is an ecologically diverse, viviparous, monophyletic order of sharks, where modern forms consist of eight families, 10 genera, and 15 species (Compagno, 2002; Stone & Shimada, 2019). There are a number of studies that examined the axial skeletal or muscular anatomy (cranium, trunk, or caudal fin) in extant lamniforms (e.g., Achebe et al., 2013; Compagno, 1990; Dolton, Jackson, et al., 2023; Dolton, Snelling, et al., 2023; Kim et al., 2013; Stone & Shimada, 2019; Wilga, 2005). On the other hand, papers exploring the appendicular anatomy (pectoral and pelvic fins) are rare (Silva et al., 2023; Tomita et al., 2014), and those particularly focusing on claspers and/or siphon sacs are extremely scant and limited to *Lamna nasus* (Bonnaterre, 1788) and *Cetorhinus maximus* (Gunnerus, 1765) (Jungersen, 1899; Matthews, 1950). In an attempt to increase information on the secondary sexual characters of Lamniformes, we investigated the anatomy of the pelvic region in a monospecific pseudocarchariid shark, *Pseudocarcharias kamoharai* (Matsubara, 1936) (crocodile shark), in detail for the first time. The species is the smallest lamniform taxon, measuring up to only 1220 mm in total length (TL). Additionally, it possesses some distinctive characteristics, such as large eyes, long and slender teeth, and small fins, and is distributed worldwide throughout the tropical ocean waters between the surface and at least 590 m in depth (Ebert et al., 2021; Reichmann et al., 2025). Based on the clasper calcification and clasper length, males from the equatorial and tropical western Atlantic Ocean are estimated to mature at around 760 mm TL, where the claspers in adults usually measure more than 80 mm in inner length (CLI) and 65 mm in outer length (CLO) (Oliveira et al., 2010). Our present study, which provides new morphological information on secondary sexual characteristics, is anticipated to offer potentially informative phylogenetic characters for future morphology‐based systematic studies of lamniforms and other elasmobranchs, as well as for comparative analyses and paleontological reconstructions of related taxa in the fossil record.

## 2 MATERIALS AND METHODS

We examined three specimens of *P. kamoharai*, that are preserved in 70% ethanol, previously fixed with formalin, and are deposited at the following two institutions: Universidade Federal da Paraíba, Brazil (UFPB), and the National Museum of Natural History, Smithsonian Institution (USNM). They consist of two males (UFPB 5355, 1000 mm TL, 852 mm in fork length [FL]; USNM 303207, 1081 mm TL, 917 mm FL) and one female specimen (UFPB 5356, 980 mm TL, 823 mm FL) (note: the TL and FL measurements follow Compagno, 1984). The skeletal and muscular anatomy of their pelvic fins and sexual structures were analyzed through manual dissections. In addition, for comparative purposes, the computed tomography (CT) scan of *L. nasus* housed in the Zoological Museum, University of Copenhagen, Denmark (ZMUC P2397040, male, ~1800 mm TL) was obtained from MorphoSource database (https://www.morphosource.org/projects/000497872?locale=en). The CT scan enabled the visualization of the pelvic fin skeleton of a mature and rare species, facilitating our discussion below. The CT scan was performed using a Toshiba Aquillon Prime SP system with the following parameters: X‐ray tube voltage = 120 kVp, x‐ray tube current = 260–450 mA, integration time = 1000 ms, spatial resolution = 0.4–1.0 mm isotropic, convolution kernel = FC18, acquisition time = 60–90 s per scan (Winkel et al., 2024).

Through manual dissection, the siphon sac of *Pseudocarcharias* was first exposed before its removal for subsequent exposure of the pelvic and clasper musculatures. Because there are no standardized measurements related to the siphon sac to our knowledge, we measured the following four variables directly from each of the male specimens examined (Figure 1): (1) the distance between the anterior margin of the siphon sac to the anterior margin of the pelvic girdle (ASP); (2) between the girdles, from the posterior margin of the pectoral girdle to the anterior margin of the pelvic girdle (DBG); (3) between the posterior margin of the pectoral girdle to the anterior margin of the siphon sac (PGS); and (4) siphon sac TL between its anterior and posterior margins (STL). The epidermis and the connective tissues were separated from the siphon sac. The width of the sac was calculated by simply measuring only the left side and then doubling it.

**FIGURE 1:**
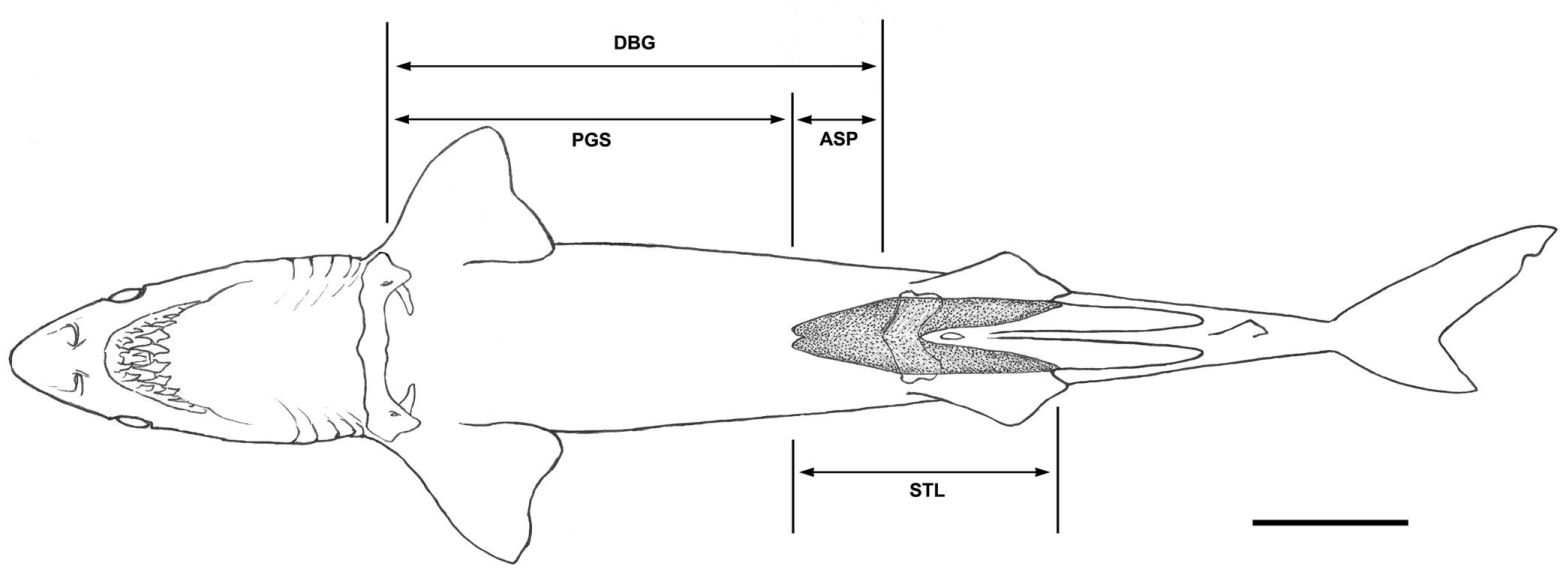
Anatomical illustration of the siphon sac (dotted) and the girdles of *Pseudocarcharias kamoharai* (UFPB 5355, male, 1000 mm TL) in ventral view. Anterior margin of siphon sac to anterior margin of the pelvic girdle (ASP), distance between girdles from the posterior margin of the pectoral girdle to the anterior margin of the pelvic girdle (DBG), posterior margin of the pectoral girdle to the anterior margin of the siphon sac (PGS), and siphon sac total length from the anterior to the posterior margins (STL). Scale bar = 100 mm.

After removing the siphon sac, the muscles and skeleton of the pelvic fins were then manually cleaned, and the anatomical elements were identified based on the published literature (e.g., Daniel, 1934; Denison, 1937; Goto et al., 1999; Macesic & Kajiura, 2010; Silva & Casas, 2020). Anatomical terminology of the clasper skeleton and musculature follows Jungersen (1899) and Matthews (1950), who both used the nomenclature for the axial cartilage as the structure formed by the appendix‐stem (or stem cartilage) and the dorsal and ventral marginal cartilages. The use of the abbreviation “ax” for appendix‐stem was preferred considering that recent papers have been using it consistently instead of the abbreviation “b” (e.g., Petean & Carvalho, 2018; Silva & Casas, 2020). The terminology for the pelvic skeleton follows Silva (2014), Silva et al. (2023), and Silva and Vaz (2023), where some previously published abbreviations were modified for clarity. Photographs were taken and edited using the software Adobe Photoshop CS.

## 3 RESULTS

### 3.1 External morphology

The male specimens have the CLO of 65–69 mm and CLI of 105–110 mm. The clasper (Figure 2a) is conical with a wider base and a tapering distal one‐quarter. It has a dark brown color like the rest of the body, but the base and the mediodorsal region have a cream color. The clasper groove (G) is prominent, the spur is evident (t3), and a rhipidion (RH) and a cover rhipidion (CHR) are present in the mediodistal part of the clasper (Figure 2b,c). The pelvic fin of the males is dark brown in color, whereas that of the females is light brown.

**FIGURE 2:**
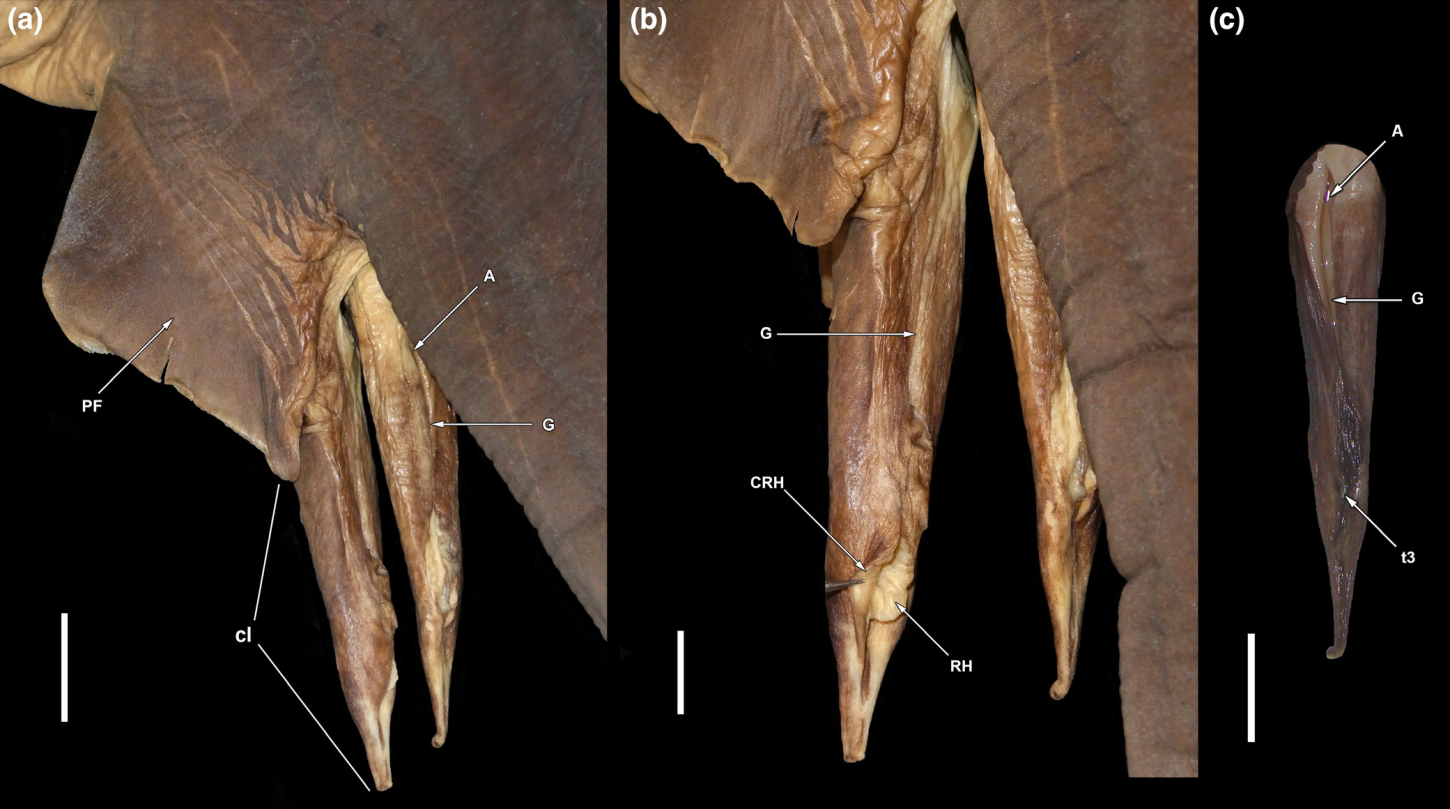
Dorsal view of the clasper and the left pelvic fin (a) and close‐up of the left clasper (b) of *Pseudocarcharias kamoharai* (USNM 303207, male, 1081 mm TL). (c) Dorsal view of the left clasper of *P. kamoharai* (UFPB 5355, male, 1000 mm TL). Apopyle (A), clasper (cl), clasper groove (G), cover rhipidion (CRH), pelvic fin (PF), rhipidion (RH), and spur (t3). Scale bars: 20 mm (a, c); 10 mm (b).

### 3.2 Siphon sac

The siphon sacs (S) overall form an inverted “Y” in shape, with an “M”‐shaped anterior margin and a forked posterior portion (Figures 3, 4). Each end of the siphon sac is associated with the left and the right clasper individually. The siphon sac is well developed and extends anteriorly more than 69–70 mm (ASP) in relation to the anterior margin of the pelvic girdle, and its anterior portion is nearly 299–300 mm (PGS) away from the posterior margin of the pectoral girdle. Additionally, its posterior region ends inside the clasper, more exactly in the apopyle. The TL of the exposed siphon sac is 139–140 mm (STL) (Figures 3, 4). The covered portion of the siphon sac is only observable after the dissection of the clasper. Both portions, the exposed (140 mm) and the covered (siphon tube, 5 mm), have a total combined length of 145 mm, where this measurement was only possible for UFPB 5355. The ASP has one‐fifth of the distance (69–70 mm) between the pectoral and pelvic girdles (359–377 mm). Its greatest width is on its midportion, which is 45–48 mm, with narrower anterior and posterior portions. A longitudinal connective tissue (CT) separates the two sacs throughout their whole anteroposterior extension that continues posteriorly separating this secondary sexual structure from the clasper musculature, as well as from the pelvic and hypaxial musculatures. Additionally, the siphon sac appears to consist of smooth muscles only, which is distinct from the surrounding skeletal musculature. The difference was made by the general observation of the fibers with the naked eye, where the bundles were not as well defined as those of the pelvic musculature.

**FIGURE 3:**
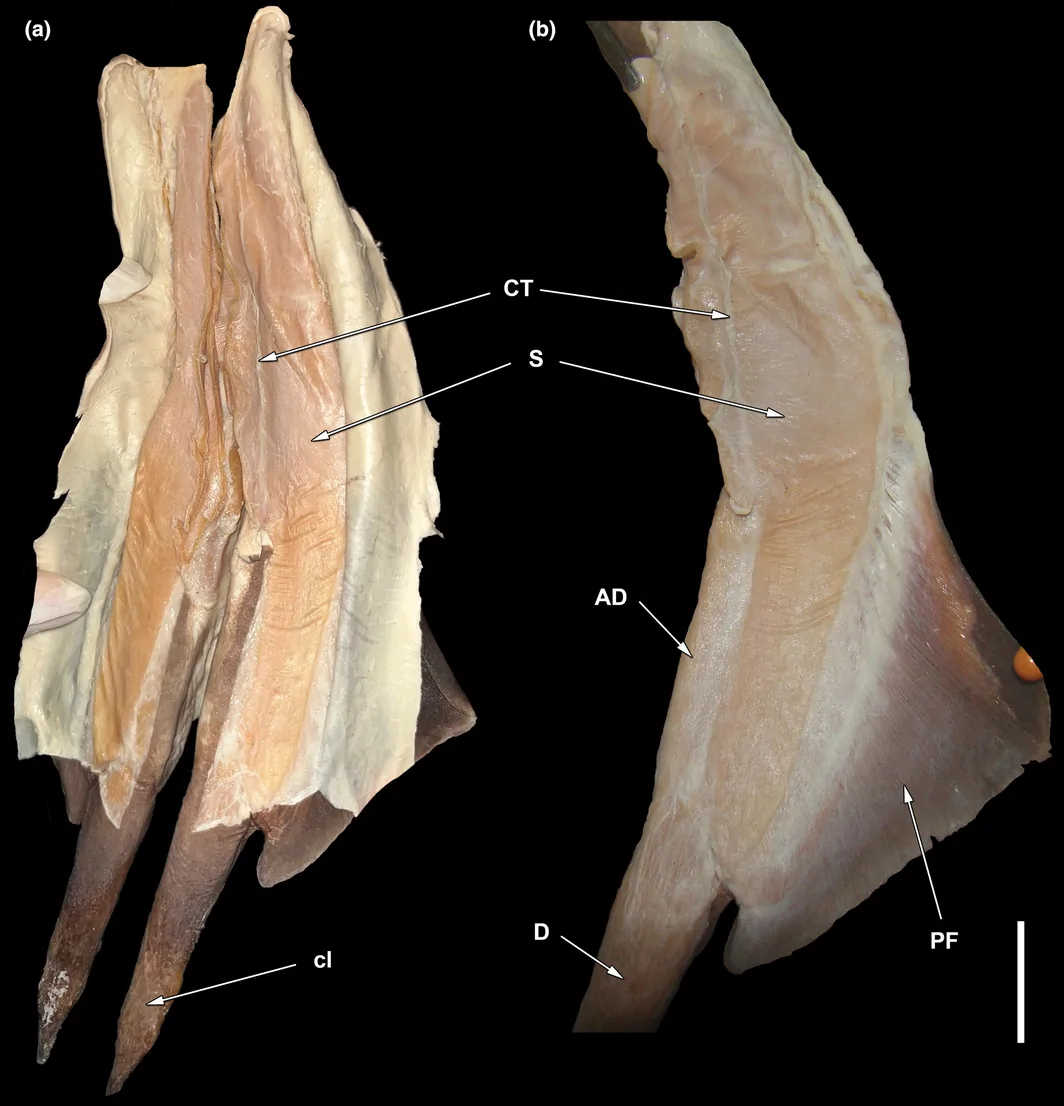
Ventral view of the siphon sacs and claspers of *Pseudocarcharias kamoharai* (UFPB 5355, male, 1000 mm TL). (a) Left and right siphon sacs. (b) Detail of the left siphon sac. *Adductor* muscle (AD), clasper (cl), connective tissue (CT), *dilatator* muscle (D), pelvic fin (PF), and siphon sac (S). Scale bar = 20 mm.

**FIGURE 4:**
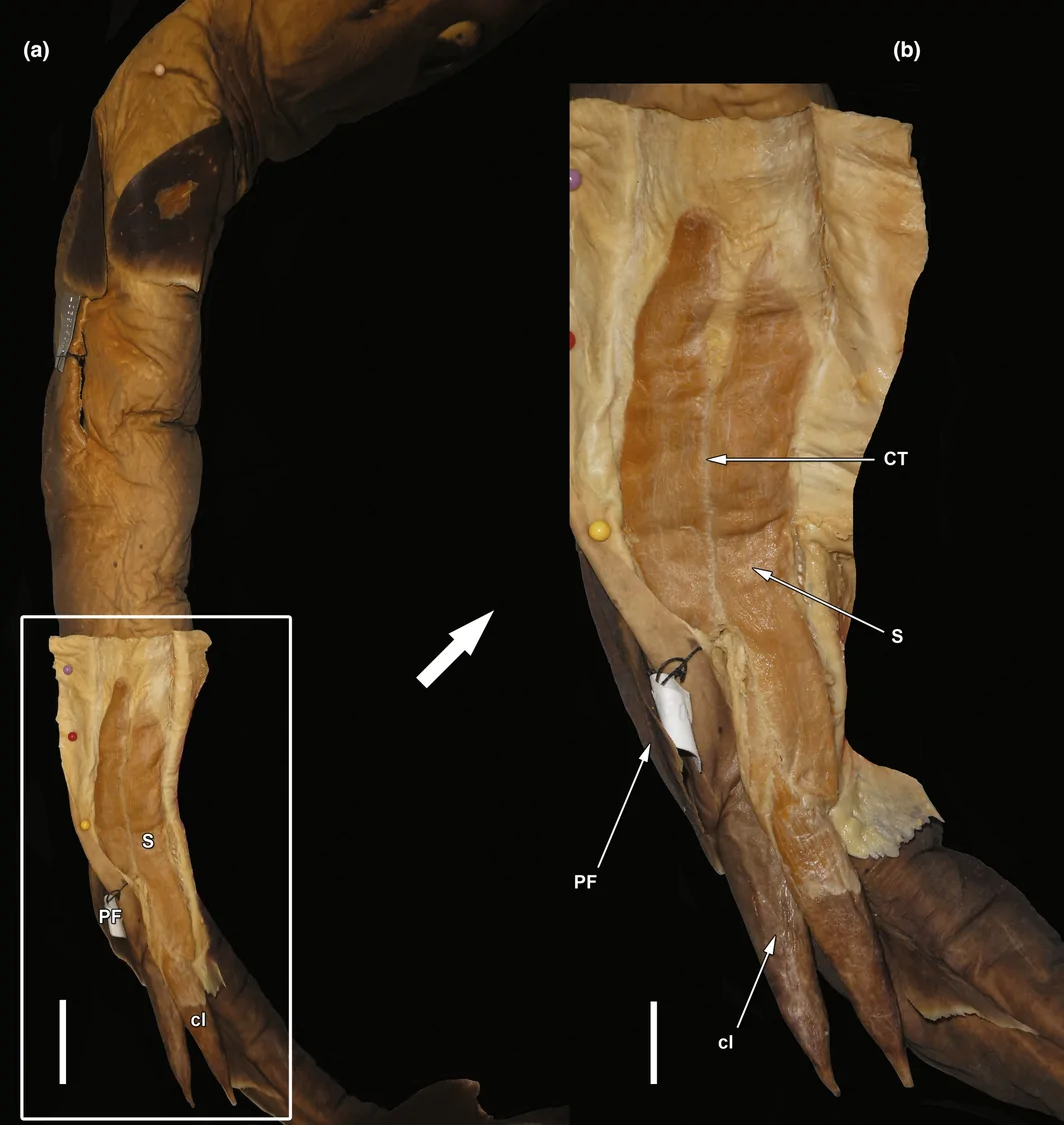
Ventral view of the siphon sacs and claspers of *Pseudocarcharias kamoharai* (USNM 303207, male, 1081 mm TL). (a) Siphon sac size in relation to body length. (b) Zoomed region of siphon sac. Clasper (cl), connective tissue (CT), pelvic fin (PF), and siphon sac (S). Scale bars: 40 mm (a); 20 mm (b).

### 3.3 Clasper skeleton

The claspers of both male specimens were mostly calcified, demonstrating that the individuals were sexually mature. Only their distal extremity was still flexible. The skeleton of the clasper of *P. kamoharai* consists of seven cartilaginous elements: appendix‐stem (ax), beta (*β*) cartilage, dorsal (dm) and ventral (vm) marginal cartilages, dorsal (dt) and ventral (vt) terminal cartilages, and a spur or third terminal cartilage (t3) (Figure 5).

**FIGURE 5:**
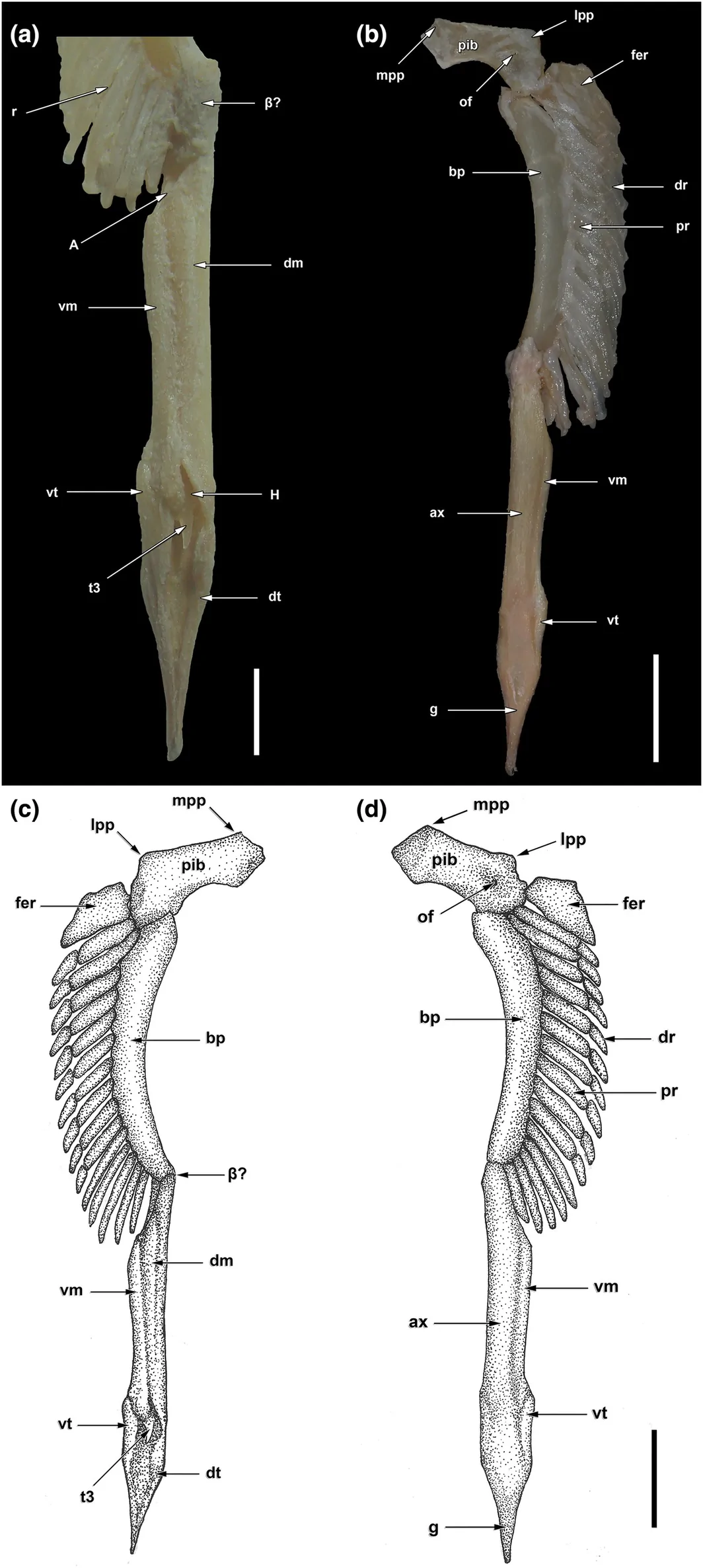
Dorsal (a, c) and ventral (b, d) views of the pelvic fin and clasper of *Pseudocarcharias kamoharai* (UFPB 5355, male, 1000 mm TL). Clasper: Apopyle (A), appendix stem (ax), beta cartilage (*ꞵ*), dorsal marginal cartilage (dm), dorsal terminal cartilage (dt), end‐style (g), hypopyle (H), spur (t3), ventral marginal cartilage (vm), and ventral terminal cartilage (vt). Pelvic fin: Anteromedian prepelvic process (mpp), basipterygium (bp), distal radial (dr), first enlarged pelvic radial (fer), lateral prepelvic process (lpp), obturator foramina (of), proximal radial (pr), puboischiadic bar (pib), and radials (r). Scale bars: 10 mm (a); 20 mm (b–d).

The appendix‐stem articulates anteriorly with the basipterygium (bp) and extends to the distal end of the clasper. The dorsal and ventral marginal cartilages are firmly articulated to the lateral edges of the appendix‐stem and form the lateral walls of the clasper groove (G), which functions as a tube. The anterior portion of this tube is the apopyle (A), whereas the distal end is known as the hypopyle (H) (Figure 5a). The dorsal marginal cartilage has a longitudinal groove, and its separation from the ventral marginal cartilage is clear (Figure 5a,c), and a small *β* cartilage may be fused with the appendix‐stem on its medial base. Although the spur or t3 is claw‐shaped and prominent on the dissected clasper, the spur is not readily visible on the non‐dissected clasper, which is in a completely relaxed state. Unlike the marginal cartilages, the terminal ones are not juxtaposed on the dorsal side of the clasper. They are connected with the end of the appendix‐stem, which is identified as the end‐style (g), which is a slightly tapered structure. The dorsal cartilages are only visible in dorsal view.

### 3.4 Pelvic fin and clasper musculature

The dorsal surface of the male pelvic fin (Figure 6a,c) has two muscles, the *levator pelvicus profundus* (LP) and *levator pelvicus superficialis* (LS). The LP originates from the lateral region of the basipterygium and inserts on the distal radials and the ceratotrichiae. On the other hand, the LS originates from the connective tissue of the hypaxial musculature and inserts on the proximal portion of the dorsal marginal cartilage, the beta cartilage, and likely on the *levator pelvicus profundus*, sharing also its points of insertion (distal radials and ceratotrichiae). Additionally, the *adductor* muscle (AD) is present on both the dorsal and ventral surfaces of the pelvic fin (Figure 6). It arises from the linea alba (LA) and a dorsal aponeurosis (AP) on the posteromedial region of the puboischiadic bar and inserts ventrally on the basipterygium and the base of the appendix‐stem (ax) and dorsally on the *β* cartilage by an aponeurosis. Laterally to the *adductor*, the *flexor internus* (FIT), a muscle commonly associated with the clasper, originates from the proximomedial portion of the basipterygium and inserts on the *β* cartilage and the proximomedial region of the ax. The *flexor internus* and the *adductor* have a superficial separation, but they form a single muscle block more deeply (Figure 6a). The presence of the *flexor externus* muscle is uncertain in the examined specimens.

**FIGURE 6:**
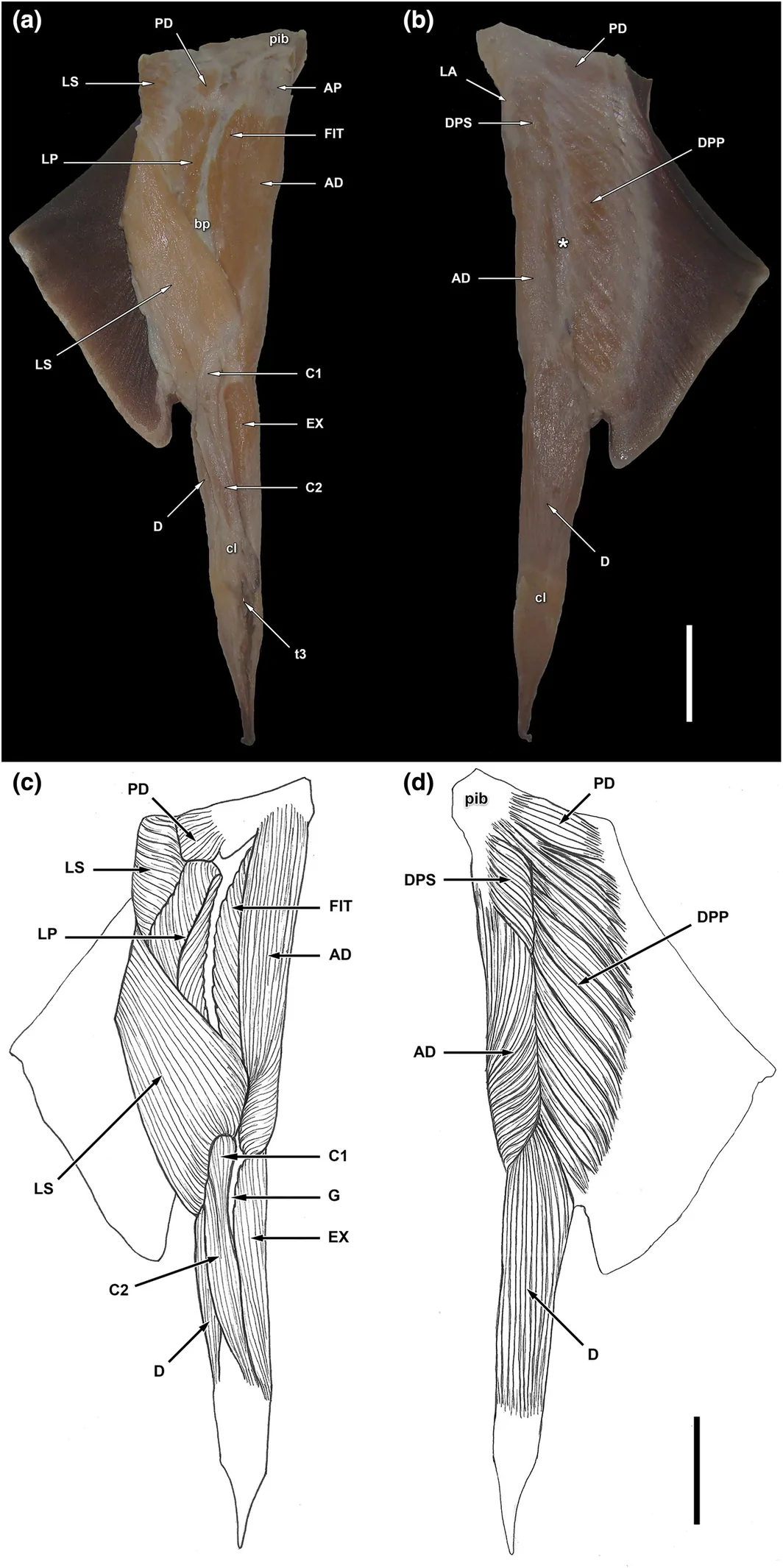
Photographs and illustrations of the dorsal (a, c) and ventral (b, d) views of the musculature of the right pelvic fin and clasper of *Pseudocarcharias kamoharai* (UFPB 5355, male, 1000 mm TL). *Adductor* (AD), aponeurosis (AP), clasper (cl), clasper groove (G), *compressor* (C1 and C2), *depressor pelvicus profundus* (DPP), *depressor pelvicus superficialis* (DPS), *dilatator* (D), *extensor* (EX), *flexor internus* (FIT), *levator pelvicus profundus* (LP), *levator pelvicus superficialis* (LS), *linea alba* (LA), *propterygium depressor* (PD), puboischiadic bar (pib), spur or third terminal cartilage (t3), and raphe of the basipterygium in asterisk (*). Scale bars = 20 mm.

The ventral musculature associated with the pelvic fin is represented by the *propterygium depressor* (PD) and *depressor pelvicus*, the latter of which is divided into two portions, a superficial one and a deep one (Figure 6b,d). The *depressor pelvicus superficialis* (DPS) is located immediately above the *adductor* and originates from the ventral posteromedian portion of the puboischiadic bar by the linea alba and inserts on the proximal portion of the raphe of the basipterygium (*). The *depressor pelvicus profundus* (DPP) originates from the raphe of the basipterygium and inserts on the distal radials (dr) and ceratotrichiae, and the two anteriormost bundles of the DPP are connected with the puboischiadic bar. Above the DPP is the *propterygium depressor*, which originates from the lateral prepelvic process (lpp) of the puboischiadic bar and inserts on the first enlarged pelvic radial (*fer*; = pelvic propterygium).

The clasper has three muscles: the *compressor* and *extensor* on the dorsal surface, and the *dilatator* covering the entire ventral surface. The *compressor* is divided into two portions, C1 and C2, and is associated with the siphon sac (Figure 6a,c). C1 is slightly oblique and is located at the anterior portion of the clasper groove, whereas C2 forms a bundle lying alongside the clasper groove. C1 originates from the *β* cartilage and inserts on the dorsal marginal cartilage. C2 originates from the dorsal marginal cartilage, medial to the groove that separates the two marginal cartilages and inserts on the posterodorsal portion of the ventral marginal cartilage (vm). Arising from the proximal portion of the dorsal marginal cartilage is the *extensor* muscle (EX), which inserts on the dorsal terminal cartilage of the clasper. The clasper groove is located between the *extensor* and C2. The large *dilatator* muscle (D) (Figure 6b,d) originates from the posterior end of the basipterygium and inserts on the proximal half of the appendix‐stem, as well as on the ventral marginal and ventral terminal cartilages on the dorsal side of the clasper. Positioned medial to the origin of this muscle is the *adductor*, which is also observable on the dorsal side of the clasper.

The organization of the pelvic muscles on the laterodorsal surface of the female is very similar to that of the male (Figure 7b), with the *levator pelvicus profundus* (LP) having the same insertions in both sexes, whereas the origin is also from the lateral of the basipterygium, in addition to the lateral portion of the puboischiadic bar. The *levator pelvicus superficialis (*LS) (Figure 7a) also originates from the fascia that covers the hypaxial musculature, but the insertion is on the distal radials and the ceratotrichiae, as observed for the *levator pelvicus profundus*. The *adductor* is present on the mid‐dorsal and midventral surfaces of the pelvic fin (Figure 7), as well as the *depressor pelvicus superficialis* (DPS). The DPS originates from the linea alba and inserts on the proximal part of the raphe of the basipterygium, whereas the *adductor* originates from the linea alba and a dorsal aponeurosis and inserts on the posterior portion of the basipterygium and on the last pelvic radial. The *adductor* muscle is positioned below the *depressor pelvicus superficialis*.

**FIGURE 7:**
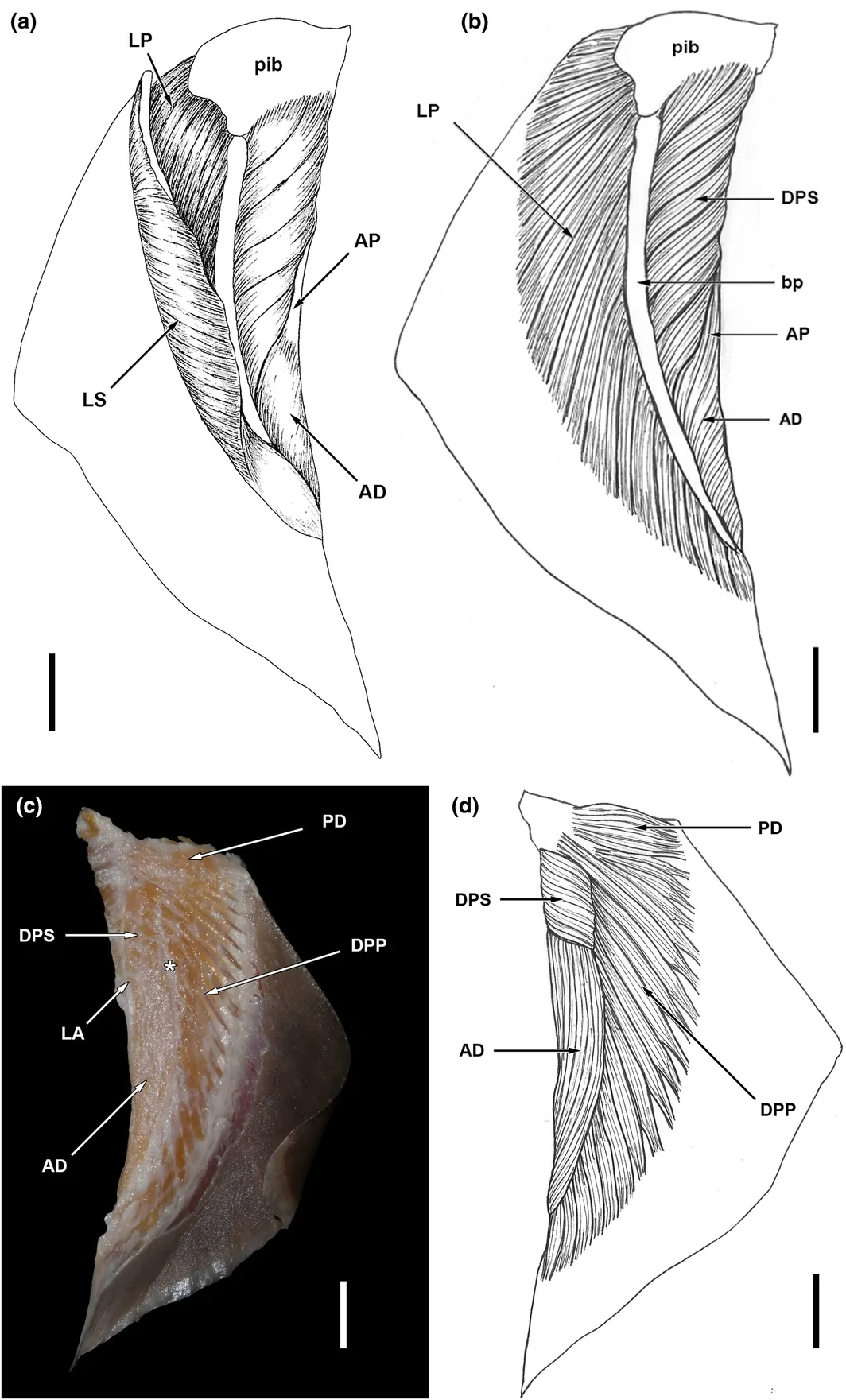
Photograph and illustrations of the dorsal (a, b), and ventral (c, d) views of the left pelvic fin of *Pseudocarcharias kamoharai* (UFPB 5356, female, 980 mm TL). In (b) the LS was removed to expose the LP. *Adductor* (AD), aponeurosis (AP), basipterygium (bp), *depressor pelvicus profundus* (DPP), *depressor pelvicus superficialis* (DPS), *levator pelvicus profundus* (LP), *levator pelvicus superficialis* (LS), *linea alba* (LA), *propterygium depressor* (PD), puboischiadic bar (pib), and raphe of the basipterygium in asterisk (*). Scale bars = 10 mm.

The female has the same pelvic muscles with a similar arrangement on the ventral surface as in the male (Figure 7c,d). Anteriorly, the *propterygium depressor* (PD) originates from the lateral prepelvic process (lpp) of the puboischiadic bar and inserts on the first enlarged pelvic radial. Immediately below is the *depressor pelvicus profundus* (DPP). It originates from the raphe of the basipteryium (*) and inserts on the distal radials and ceratotrichiae. As in males, the two anteriormost bundles of the DPP are connected with the puboischiadic bar. Furthermore, all the ventral muscles (PD, DPS, DPP, and *adductor* [AD]) and some of the dorsal ones (LP and LS) are found both in males and females. The muscles found exclusively in males are: the *flexor internus* (FIT), *compressor* muscles (C1 and C2), *dilatator* (D), and *extensor* muscle (EX), which seems to be related with the presence of an intromittent organ, the clasper.

## 4 DISCUSSION

### 4.1 Siphon sac

The siphon sac is an understudied sexual structure, even though Mianutti's (2025) thesis recently established the primary homology of the sac for all chondrichthyans and verified the differences and similarities within the group. Overall, *P. kamoharai*, which was the only lamniform taxon included in Mianutti's (2025) study, shares some morphological aspects of the siphon sac with most chondrichthyans. For example, the sacs are over the pelvic fins, they extend beyond the pelvic girdle, and they are united by a connective tissue (Mianutti, 2025). Within the order Lamniformes, *L. nasus* (porbeagle shark) and *C. maximus* (basking shark) are the only species in which the pelvic fin morphology was previously explored, but no illustrations of the sacs were presented in the studies (Jungersen, 1899; Matthews, 1950). Leigh‐Sharpe (1921, 1926) stated that *L. nasus* has a small siphon sac and a clasper gland occupying practically the entire space of the sac. This is an interesting characteristic given that the presence of a clasper gland inside the sac is a condition usually found in batoids and *Squatina* (Jungersen, 1899; Leigh‐Sharpe, 1926; Moreira & Carvalho, 2019). Matthews (1950) also described a clasper gland in *C. maximus*, even though its location and morphology were different from *L. nasus*, where it was not inside the siphon sac, but rather within the ventral marginal cartilage facing the duct of the clasper. Matthews (1950) added that this type of clasper gland was briefly noted once for a batoid species, *Dipturus batis* (Linnaeus, 1758), but such a condition was never described before in the literature for any elasmobranch. However, no clasper gland was found in *P. kamoharai*.

The morphology of the siphon sac of *C. maximus* was described as wider at the anterior end rather than at its midportion (Matthews, 1950), a condition contrary to *P. kamoharai* having the greatest width on the midportion of the sac. The TL of the siphon sac was 1360 mm, and the distance from the base of the pectoral fins to the anterior portion of the siphon sac was 600 mm (Matthews, 1950); yet, there was also a small portion of the sac (50 mm) extending inside the clasper, the siphon tube. Additionally, the distance of the base of the pectoral girdle to the beginning of the siphon sac (PGS) was described as nearly two times the distance (Matthews, 1950) of that observed in *P. kamoharai* in the present study. Nevertheless, Matthews (1950) did not provide the distance between the pectoral and pelvic girdles, so it is not possible to relate the size of the siphon sac to this distance. However, when comparing the distance between the pectoral girdle and the beginning of the siphon sac in relation to the overall length of the siphon sac, it is clear that *P. kamoharai* has a shorter siphon sac (with nearly half of the distance between the pectoral girdle and the beginning of the sac), whereas it is longer in *C. maximus* (more than two times the distance of the pectoral girdle to the beginning of the sac). The siphon sac in *L. nasus* (or the bag‐shaped part of the *compressor* muscle as reported by Jungersen, 1899) is positioned 230 mm above the pelvic fins, nearly three times that of *P. kamoharai* (77 mm), and has 400 mm of total length, almost three times the length observed in *P. kamoharai* (with 139–140 mm).

### 4.2 Components of the pelvic fin and clasper musculature

The only two papers that analyzed the skeleton and musculature of the claspers in Lamniformes (Jungersen, 1899; Matthews, 1950) are very incomplete regarding the musculature associated with the pelvic fins. The same applies to the works of Denison (1937), Daniel (1934), and Gilbert and Heath (1972), who only investigated the *flexor* muscles of the pelvic fins. All of them were inadequate when identifying the pelvic muscles by referring to them in a general way, either simply as radial muscles or ray muscles. On the other hand, Goto et al. (1999) recognized three muscles of the pelvic fin in a female of *Hemiscyllium ocellatum* (Bonnaterre, 1788; Orectolobiformes): the *levator*, *depressor* and *adductor pelvicus*. These muscles were also identified in *P. kamoharai*, although another pelvic muscle was identified in addition to those three: that is, the PD that was previously reported by Macesic and Kajiura (2010) in batoids. Based on Macesic and Kajiura's (2010) work, we were also able to identify a subdivision of the *depressor* and *levator pelvicus* in distal (*profundus*) and proximal (*superficialis*) regions. It is important to highlight that the musculature of batoids is usually more complex than that of sharks, especially of benthic species. However, we were able to identify the remaining pelvic fin muscles in *P. kamoharai* when comparing its origin and insertion with those of batoids.

#### 4.2.1 Pelvic fin musculature

In relation to the musculature of the pelvic fin, the *adductor* originates from the basipterygium in both *L. nasus* and *C. maximus*. However, in *P. kamoharai*, it originates from the dorsal aponeurosis on the posteromedial portion of the puboischiadic bar and from the linea alba. The insertion of this muscle in *C. maximus* is on the base of the axial cartilage whereas it is on the appendix‐stem, the ventral surface of the basipterygium, and the beta cartilage in *P. kamoharai*. This muscle was not fully examined for *Lamna* (Jungersen, 1899).

Goto et al. (1999) and Goto (2001), who analyzed the pelvic musculature in orectolobiforms, appears to have mistakenly identified the *depressor pelvicus superficialis* (DPS) as the *adductor pelvicus* (“apv”) and the *depressor pelvicus profundus* (DPP) only as the *depressor pelvicus* (“dpv”). The *adductor* would be in the medial portion of the pelvic fin, but this muscle is not depicted in the illustrations of his works (Goto et al. 1999, fig. 3; Goto, 2001, fig. 65). His “apv” has the same origin and insertion of the muscle DPS in *P. kamoharai* (this study) as well as in batoids (Macesic & Kajiura, 2010). Moreover, Goto et al. (1999) and Goto (2001) considered the *depressor pelvicus* as a muscle that would be subdivided into two layers: an anterolateral and a posterolateral. However, we disagree with this interpretation, where Goto et al. (1999) and Goto (2001) likely missed recognizing the presence of the *propterygium depressor*. Our interpretation is supported by the fact that the DPS has the same sites of origin and insertion in *P. kamoharai* and in the distantly related group of batoids (Macesic & Kajiura, 2010). Denison (1937) also seems to have misidentified the DPS for the *adductor* and the *adductor* for the *flexor externus*, based on the position of these muscles, where the *adductor* generally represents the medialmost muscle. This is illustrated by Denison (1937) through the features of the “*adductor*” (this muscle is visible on both surfaces [ventral and dorsal], and ventrally each bundle is well defined and originates from the pelvic girdle, whereas it inserts mostly on the basipterygium, as the DPS in the crocodile shark) and the features of the “*flexor externus*” (it originates from a broad flat tendon from the pelvic girdle near the midline and inserts on the beta cartilage and the appendix‐stem, similar to the *adductor* of *P. kamoharai*).

*Pseudocarcharias kamoharai* has different sites of origin and insertion for the muscle *levator pelvicus* compared to that of *H. ocellatum* (Goto et al., 1999) and benthic batoids (Macesic & Kajiura, 2010). Goto et al. (1999) found only one *levator pelvicus* for *H. ocellatum*, and it has the same origin as the *levator pelvicus superfficialis* (LS) in *P. kamoharai*, which is the fascia of the hypaxial musculature. Its insertion is on the dorsal surface of the basipterygium and the radials, similar in part to the condition observed for the *levator pelvicus profundus* (distal radials and ceratotrichiae) of *P. kamoharai*. Conversely, what was identified as the posterodorsal portion of the “muscles of the pelvic fin” in *C. maximus* by Matthews (1950) is interpreted to be part of the muscle LS in *P. kamoharai*.

Additionally, the muscle “*general propterygium depressor”* is found in *P. kamoharai* and in benthic batoids (Macesic & Kajiura, 2010), with some differences in relation to the sites of origin and insertion in *Pseudocarcharias*. However, we prefer to refer to it as the PD for *P. kamoharai*, considering that the rays possess three different muscles that form the *propterygium depressor*, and it would be premature to define if only the *general* PD is present in *P. kamoharai*. Many of the differences between batoids and sharks are related to their distinct body structures (e.g., the presence of the iliac process on the puboischiadic bar and the anterior extension of the propterygium in batoids: Silva, 2014; Silva et al., 2021; Silva & Vaz, 2023). The PD originates from the iliac process of the puboischiadic bar in batoids, whereas in *P. kamoharai* it originates from the lateral prepelvic process of the pelvic girdle. Nevertheless, the insertion is the same, which is at the first enlarged pelvic radial.

#### 4.2.2 Clasper musculature

In relation to the musculature of the clasper, the origin and insertion of the *dilatator* muscle of *P. kamoharai* is similar to the one described for *C. maximus* (Mathews 1950). Both of them originate from the distal end of the basipterygium and insert on the proximal half of the appendix‐stem. Yet, in *P. kamoharai*, this muscle also inserts on the ventral marginal and terminal cartilages. On the other hand, the origin and insertion of the *extensor* is different in these species. In *C. maximus*, it inserts onto the medial side of the appendix‐stem, whereas it inserts on the dorsal marginal cartilage in *P. kamoharai*. There is no information in the literature regarding the *dilatator* or the *extensor* muscles in *L. nasus* (Jungersen, 1899).

The *compressor* muscle of *P. kamoharai* is formed by two different bundles (C1 and C2). Jungersen (1899) named one of the components of the *compressor* as the *outer lip‐muscle*, which we interpret as homologous to the C2. However, Jungersen (1899) did not describe the origin and insertion of this muscle. Conversely, Matthews (1950) indicated only the insertion of the C2, which is on the ventral marginal cartilage, mostly on the base of the spur. In *P. kamoharai*, the insertion of the C2 is only on the posterodorsal portion of the ventral marginal cartilage. Jungersen (1899) did not describe the C1, and Matthews (1950) did not explore this musculature.

The *compressor* muscle was examined in more detail in the broadnose sevengill shark, *Notorynchus cepedianus* (Péron, 1807), by Daniel (1934). He described the origin of the *compressor* muscle as being at the beta cartilage, whereas its insertion was on the last radial cartilage, the same origin found for the muscle C1 in *P. kamoharai*. Daniel (1934, fig. 96) also mentions a “muscle of sac” in his illustration without noting its origin and insertion, which we have interpreted to be equivalent to the C2 in *P. kamoharai*.

In *P. kamoharai*, only the *flexor internus* could be identified, although it is uncertain if the *flexor externus* would be fused to this muscle. Additionally, no information about these muscles was provided for other lamniforms in the literature (Jungersen, 1899; Matthews, 1950). According to our analysis, the dorsal posterolateral portion of the muscle *adductor* in *C. maximus* (Matthews, 1950) seems to be the *flexor internus* in *P. kamoharai*. Both *adductor* (*C. maximus*) and *flexor internus* (*P. kamoharai*) originate on the basipterygium and insert on portions of the clasper skeleton (= beta cartilage) in *P. kamoharai* and the base of the axial cartilage (= the appendix‐stem and both marginal cartilages) in *C. maximus*.

The *flexor internus* of *P. kamoharai* is similar to the origins and insertions of both *flexors* (*flexor internus* and *externus*) in *N. cepedianus* (Daniel, 1934). All three muscles, *flexor internus* of *P. kamoharai* and *flexor internus* and *externus* of the *N. cepedianus*, originate from the basipterygium; however, in *N. cepedianus*, the *flexor externus* originates specifically from the proximomedial portion of the basipterygium, similar to the condition observed for the *flexor internus* in *P. kamoharai*. Additionally, the *flexor internus* in *P. kamoharai* inserts on the beta cartilage and the proximomedial region of the appendix‐stem, which are the same points of insertion of the *flexor externus* and *flexor internus*, respectively, in *N. cepedianus*. In the whale shark, *Rhincodon typus* (Smith, 1828), the *flexor internus* originates from the distal‐half portion of the basipterygium and inserts on the beta cartilage (Denison, 1937). Conversely, in other orectolobiforms, the insertion of the *flexor internus* is on the medial and dorsal surfaces of the axial cartilage (Goto, 2001), whereas its origin is the same as that observed in *R. typus* and *P. kamoharai* (Denison, 1937; this study).

### 4.3 Comparisons of pelvic fin and clasper skeleton within Lamniformes

The examined male specimens exhibited well‐calcified clasper skeletons, indicating sexual maturity. The only notable exception was its distal end being slightly flexible, a condition that was also reported for *C. maximus* and *L. nasus*; although, in *L. nasus*, the appendix‐stem and the marginal cartilages are more elongated compared with *P. kamoharai* and *C. maximus* (Jungersen, 1899; Matthews, 1950). Nevertheless, the ventral elements of the clasper in *Pseudocarcharias* are different from those of *Cetorhinus* and *L. nasus*. In *C. maximus* and *L. nasus*, the ventral and dorsal marginal cartilages and the ventral and dorsal terminal cartilages are visible in ventral view. On the other hand, only the ventral cartilages are visible in ventral view for *P. kamoharai*. A prominent slit is present within the distal portion of the marginal cartilages in *L. nasus* (Jungersen, 1899, fig. 22; Figure 8b). This space is narrow and barely observable in *C. maximus* (Matthews, 1950), and the slit is even absent in *P. kamoharai*.

**FIGURE 8:**
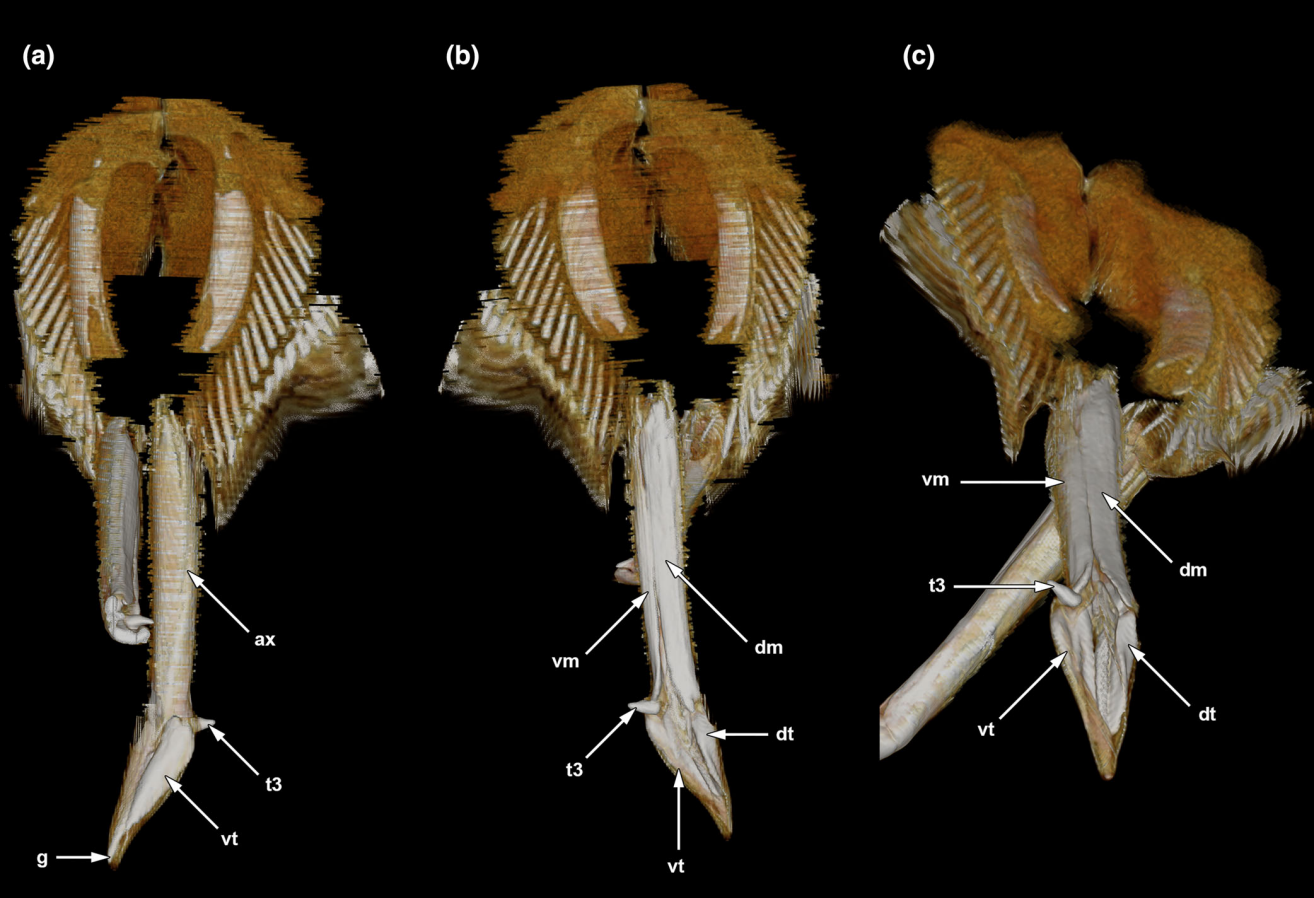
CT scan of the pelvic fins and claspers of *Lamna nasus* (ZMUC P2397040, male, ~1800 mm TL) in ventral (a), dorsal (b), and oblique (c) views. Clasper: Appendix‐stem (ax), dorsal marginal cartilage (dm), dorsal terminal cartilage (dt), end‐style (g), spur (t3), ventral marginal cartilage (vm), and ventral terminal cartilage (vt).

Different species of chondrichthyans seem to have at least one of the intermediate segments (b_1_, b_2_) or the beta cartilage present, with most of the shark species having both (Compagno, 1988; Daniel, 1934; Denison, 1937; Goto, 2001; Jungersen, 1899; Matthews, 1950; Petean & Carvalho, 2018). For example, the beta cartilage is present in *C. maximus*, and *L. nasus* possesses both the beta and b_1_ cartilages, which are visible in dorsal and ventral views, respectively (Jungersen, 1899; Matthews, 1950). Therefore, even though we could not confirm the presence of the beta cartilage in *P. kamoharai* likely because of the small size of the body (vs. *L. nasus* and *C. maximus*, that had two or even nine times the size of *P. kamoharai*), it may also be present in this species. In *L. nasus*, the beta cartilage is very discernible dorsally, whereas in *C. maximus*, it is subtle.

The length of the terminal cartilages varies among the three lamniform species. In *L. nasus*, all the terminal cartilages are shorter than the end‐style, which corresponds to the distal portion of the appendix‐stem (Jungersen, 1899; Figure 8). In *C. maximus*, only the dorsal terminal cartilage is smaller than the end‐style, whereas the ventral terminal is as long as this structure (Matthews, 1950). In *P. kamoharai*, both terminal cartilages extend to the posterior end of the end‐style.

Among the three lamniform taxa, *L. nasus* is distinct from the other two species (*P. kamoharai* and *C. maximus*). It is the only one with four terminal cartilages and a second ventral terminal cartilage (tv_2_) that is described as a long and thin calcified or poorly calcified lamella (Jungersen, 1899). Although the second dorsal terminal cartilage (td2) was not identified in *P. kamoharai*, its presence was noted in *L. nasus* with its length as long as the dorsal terminal cartilage and in *C. maximus* with the same size as its spur (Jungersen, 1899; Matthews, 1950). The spur apparently has the same size (proportionate to the body), shape, and location in these three lamniform species.

### 4.4 Sexual dimorphism in pelvic fin musculature

In dorsal view, the *levator pelvicus profundus* has an additional point of origin in the female, the lateral portion of the puboisquiadic bar, besides the lateral region of the basipterygium found in both sexes. However, the *levator pelvicus superficialis* has the same points of origin and insertion in the female and male. Similarly, in relation to the ventral surface of the pelvic fin, the *depressor pelvicus superficialis*, the *depressor pelvicus profundus*, and the *propterygium depressor* have the same origin and insertion in both sexes. However, although the *adductor* has the same origin in both sexes, it is noteworthy that its insertion is slightly sexually dimorphic. In the male, it inserts on the appendix‐stem and the beta cartilage of the clasper dorsally, whereas it is on the last pelvic radial in the female. More significantly, we note one other evident sexual dimorphism among elasmobranchs: that is, the presence of one additional component of the pelvic fin musculature in males, the *flexor internus* (possibly the *flexor externus* as well), in addition to the muscles of the clasper (*dilatator*, *compressor* and *extensor*). So far, this sexual dimorphism has been found only in *Squalus acanthias* Linnaeus, 1758 (Marinelli & Strenger, 1954), as a consequence to the lack of papers studying the pelvic fin musculature. Although this study placed more emphasis on the male pelvic fin musculature in *P. kamoharai* with an appreciably small sample size, this sexual dimorphism is likely present even more broadly among elasmobranchs beyond the taxa for which pelvic fin anatomy has been examined to date.

### 4.5 Concluding remarks

The morphology of the pelvic fin in sharks has been a challenging topic to address because the pelvic fin, siphon sac, and clasper musculatures have been poorly explored in the literature. Nevertheless, Jungersen (1899) analyzed the pelvic fin musculature in some elasmobranchs, and Davidoff (1879) in Holocephali (*Chimaera monstrosa* Linnaeus, 1758). Furthermore, the pelvic fin musculature has been examined in *R. typus*, *H. ocellatum*, *C. maximus*, *S. acanthias*, *Centroscyllium excelsum* Shirai and Nakaya, 1990, *Isistius brasiliensis* (Quoy and Gaimard, 1824) *Urobatis jamaicensis* (Cuvier, 1816) *Psammobatis* spp., *Potamotrygon marquesi* Silva and Loboda, 2019 other species of rays and *Callorhinchus milii* Bory de Saint‐Vincent, 1823 (Denison, 1937; Goto et al., 1999; Lamarca, 1964; Lucifora & Vassallo, 2002; Macesic & Kajiura, 2010; Marinelli & Strenger, 1954; Matthews, 1950; Moreira & Carvalho, 2018; Pears et al., 2022; Petean & Carvalho, 2018; Shirai, 1992; Silva & Casas, 2020). Yet, in relation to works involving the pelvic fin skeleton (Silva et al., 2021; Silva & Vaz, 2023), studies exploring the morphology and homology of the pelvic fin musculature in chondrichthyans remain scarce. Therefore, we hope our present study, that focuses on a lamniform taxon (*P. kamoharai*) in depth, would provide a useful comparative framework for future works on the subject.

## AUTHOR CONTRIBUTIONS

**Laura F. Mianutti:** Conceptualization; investigation; methodology; data curation; formal analysis; writing – original draft; writing – review and editing; funding acquisition. **Kenshu Shimada:** Writing – review and editing. **João Paulo Capretz Batista da Silva:** Writing – review and editing; funding acquisition; formal analysis; data curation.

## FUNDING INFORMATION

This study was funded by CNPq (no. 130844/2023‐3), CAPES (no. 88887.912453/2023‐00), Fellowship Research Award in Vertebrate Zoology Fishes (USNM—Smithsonian Institution), Fundação de Amparo à Pesquisa do Estado de São Paulo (FAPESP) (2010/52677‐6 and 2012/22692‐9), and Public Call n. 03 Produtividade em Pesquisa PROPESQ/PRPG/UFPB proposal code PIA13484‐2020.

## CONFLICT OF INTEREST STATEMENT

The authors declare no conflict of interest.

## Data Availability

All data are presented in the manuscript.
